# Trichloroethylene and Cardiac Malformations

**DOI:** 10.1289/ehp.112-a607b

**Published:** 2004-08

**Authors:** Bryan D. Hardin, Bruce J. Kelman, Robert L. Brent

**Affiliations:** GlobalTox, Inc., Hilton Head, South Carolina, E-mail: bhardin@globaltox.com; GlobalTox, Inc., Redmond, Washington; Alfred I. DuPont Institute, Wilmington, Delaware

In a report of cardiac malformations in rats exposed to trichloroethylene (TCE) in drinking water, Johnson et al. (2003) used two (1.5 and 1,100 ppm) of the four treatment concentrations that they reported in a previous study (Dawson et al. 1993). To evaluate consistency of results in this single laboratory across the 10-year interval, we compared cardiac defects reported in 2003 by Johnson et al. with those reported in 1993 by Dawson et al. Data from the two papers are shown in Table 1.

Dawson et al. (1993) did not report the number of litters per group, so that correlation was not possible. Regardless, it would be an astonishing coincidence for two studies to produce exactly the same number of fetuses in each group. Still more astonishing is the identical number of “abnormal hearts.” Nothing reported by Johnson et al. (2003) gives notice that previously published data are being reported again, but that seems to be the inescapable conclusion. If this is a republication of 1993 data, then there has also been reclassification of “defects” with the passage of time.

Another feature of the article by Johnson et al. (2003) that attracted our attention was the uncharacteristically large control group (55 litters). One can surmise that in the earlier study (Dawson et al. 1993), each group would have consisted of approximately 10 females, which is consistent with the size of exposed groups (9–13) reported by Johnson et al. Their control group, however, was unprecedentedly large, both in the context of conventional study design and relative to the other groups in this study. Johnson et al. (2003) provided no rationale for designing their study with a concurrent control five times larger than the treatment groups, which leads us to ask whether the control group reported here is, in fact, a composite of controls from multiple, perhaps five, different studies. The immediate impact of this large control group is that the very cardiac “abnormalities” at the 1.5 ppm dose that did not differ significantly from controls in 1993 become statistically significant in 2003.

Conventional developmental and reproductive toxicology assays in mice, rats, and rabbits consistently fail to find adverse effects of TCE on fertility or embryonic development aside from embryo- or fetotoxicity associated with maternal toxicity [Cosby and Dukelow 1992; Dorfmueller et al. 1979; Hardin et al. 1981; Healy et al. 1982; Manson et al. 1984; National Toxicology Program (NTP) 1985, 1986; Schwetz et al. 1975]. Johnson and Dawson, with their collaborators, are alone in reporting that TCE is a “specific” cardiac teratogen (Dawson et al. 1990, 1993; Goldberg et al. 1992; Johnson et al. 1998; Johnson et al. 2003; Loeber et al. 1988). We have always considered those findings suspect, and our comparison of data from the studies of Dawson et al. (1993) and Johnson et al. (2003) serves only to intensify our reservations. Studies from this group have potential for important public health and public policy implications, so it is particularly important for the scientific and regulatory communities to have confidence in the conduct and reporting of those studies.

We are also concerned that S.J. Goldberg, one of the authors of the publications alleging that TCE is a selective cardiac teratogen, has been a plaintiff expert in TCE lawsuits and failed to reveal that fact in his publications.

## Figures and Tables

**Table 1:** Cardiac malformations in rats exposed throughout pregnancy to drinking water containing 1.5 or 1,100 ppm TCE.

	TCE dose			TCE dose	
Cardiac abnormalities*^a^* (Dawson et al. 1993)	1.5 ppm	1,100 ppm	Heart malformations*^b^* (Johnson et al. 2003)	1.5 ppm	1,100 ppm
L-Transposition (left chest)	1	0	Abnormal looping	2	0
Great vessel defect	1	0	Aortic hypoplasia	1	0
Pulmonary valve defect	1	0	Pulmonary artery hypoplasia	1	0
Atrial septal defect	4	7	Atrial septal defect	4	7
Ventricular septal defects			Ventricular septal defects		
Subaortic	2	1	Perimembranous (subaortic)	3	3
Muscular	1	4	Muscular	1	1
Endocardial cushion defect	0	1	Atrioventricular septal defect	0	1
Aortic valve defect	0	2	Aortic valve defect	0	2
No. with abnormal hearts	9	11		9	11
No. fetuses examined	181	105		181	105

^a^Data from Dawson et al. (1993) Tables 1 and 3, Groups III and IV.

^b^Data from Johnson et al. (2003) Table 2; percentage was converted to number.

## References

[b1-ehp0112-a0607b] Cosby NC, Dukelow WR (1992). Toxicology of maternally ingested trichloroethylene (TCE) on embryonal and fetal development in mice and of TCE metabolites on in vitro fertilization. Fundam Appl Toxicol.

[b2-ehp0112-a0607b] Dawson BV, Johnson PD, Goldberg SJ, Ulreich JB (1990). Cardiac teratogenesis of trichloroethylene and dichloro-ethylene in a mammalian model. J Am Coll Cardiol.

[b3-ehp0112-a0607b] Dawson BV, Johnson PD, Goldberg SJ, Ulreich JB (1993). Cardiac teratogenesis of halogenated hydrocarbon-contaminated drinking water. J Am Coll Cardiol.

[b4-ehp0112-a0607b] Dorfmueller MA, Henne SP, York RG, Bornschein RL, Manson JM (1979). Evaluation of teratogenicity and behavioral toxicity with inhalation exposure of maternal rats to trichloroethylene. Toxicology.

[b5-ehp0112-a0607b] Goldberg SJ, Dawson BV, Johnson PD, Hoyme HE, Ulreich JB (1992). Cardiac teratogenicity of dichloroethylene in a chick model. Pediatr Res.

[b6-ehp0112-a0607b] Hardin BD, Bond GP, Sikov MR, Andrew FD, Beliles RP, Niemeier RW (1981). Testing of selected workplace chemicals for teratogenic potential. Scand J Work Environ Health.

[b7-ehp0112-a0607b] Healy TE, Poole TR, Hopper A (1982). Rat fetal development and maternal exposure to trichloroethylene 100 p.p.m. Br J Anaesth.

[b8-ehp0112-a0607b] Johnson PD, Dawson BV, Goldberg SJ (1998). A review: trichloroethylene metabolites: potential cardiac teratogens. Environ Health Perspect.

[b9-ehp0112-a0607b] Johnson PD, Goldberg SJ, Mays MZ, Dawson BV (2003). Threshold of trichloroethylene contamination in maternal drinking waters affecting fetal heart development in the rat. Environ Health Perspect.

[b10-ehp0112-a0607b] Loeber CP, Hendrix MJ, Diez De Pinos S, Goldberg SJ (1988). Trichloroethylene: a cardiac teratogen in developing chick embryos. Pediatr Res.

[b11-ehp0112-a0607b] Manson JM, Murphy M, Richdale N, Smith MK (1984). Effects of oral exposure to trichloroethylene on female reproductive function. Toxicology.

[b12-ehp0112-a0607b] NTP 1985. Trichloroethylene (CAS # 79-01-6): Reproduction and Fertility Assessment in CD-1 Mice When Administered in the Feed. NTP Report RACB84113. Research Triangle Park, NC:National Toxicology Program.

[b13-ehp0112-a0607b] NTP 1986. Trichloroethylene (CAS # 79-01-6): Reproduction and Fertility Assessment in F344 Rats When Administered in Feed. NTP Report RACB84112. Research Triangle Park, NC:National Toxicology Program.

[b14-ehp0112-a0607b] Schwetz BA, Leong KJ, Gehring PJ (1975). The effect of maternally inhaled trichloroethylene, perchloroethylene, methyl chloroform, and methylene chloride on embryonal and fetal development in mice and rats. Toxicol Appl Pharmacol.

